# Recipe for a Busy Bee: MicroRNAs in Honey Bee Caste Determination

**DOI:** 10.1371/journal.pone.0081661

**Published:** 2013-12-11

**Authors:** Xiangqian Guo, Songkun Su, Geir Skogerboe, Shuanjin Dai, Wenfeng Li, Zhiguo Li, Fang Liu, Ruifeng Ni, Yu Guo, Shenglu Chen, Shaowu Zhang, Runsheng Chen

**Affiliations:** 1 Bioinformatics Laboratory and National Laboratory of Biomacromolecules, Institute of Biophysics, Chinese Academy of Sciences, Beijing, China; 2 College of Animal Sciences, Zhejiang University, Hangzhou, China; 3 Graduate School of the Chinese Academy of Sciences, Beijing, China; 4 Research School of Biology, The Australian National University, Canberra, Australia; 5 College of Bee Science, Fujian Agriculture and Forestry University, Fuzhou, China; Monash University, Australia

## Abstract

Social caste determination in the honey bee is assumed to be determined by the dietary status of the young larvae and translated into physiological and epigenetic changes through nutrient-sensing pathways. We have employed Illumina/Solexa sequencing to examine the small RNA content in the bee larval food, and show that worker jelly is enriched in miRNA complexity and abundance relative to royal jelly. The miRNA levels in worker jelly were 7–215 fold higher than in royal jelly, and both jellies showed dynamic changes in miRNA content during the 4^th^ to 6^th^ day of larval development. Adding specific miRNAs to royal jelly elicited significant changes in queen larval mRNA expression and morphological characters of the emerging adult queen bee. We propose that miRNAs in the nurse bee secretions constitute an additional element in the regulatory control of caste determination in the honey bee.

## Introduction

Eusocial insects, of which the honey bee is the most extensively/intensively researched species, are unusual in the sense that the female exists as two (or in some cases, several) phenotypes derived from the same genotypic background [1]. In the honey bee, the worker is designed for and carries out, most functions normally assigned to motherhood, such as nest-building, feeding and caring for the brood, guarding and foraging. In essence, the worker bee is everything a mother bee should be – with the sole exception of being exactly that – a mother bee. That function is occupied by the single queen bee, who receives all the additional genomic input the colony requires by mating with a number of males, and thereafter devotes her to laying all the eggs needed to maintain the colony.

Female caste determination has traditionally been ascribed to special properties of royal jelly, which is fed in copious amounts to prospective queen bee larvae, thereby ensuring attainment of the royal status, whereas the less sophisticated diet enjoyed by the rest of the brood leads to the worker bee fate [2]. Careful analysis of the royal jelly [3], [4], [5], [6], [7], [8] has failed to identify any specific, non-nutritional “queen-making” factor, and the prevailing view is that nutrient-sensing pathways [9], [10], [11], [12] translate the dietary status of the larvae into differences in physiology and gene expression [13], [14], [15], [16], [17], [18] that are ultimately fixed by epigenetic modifications of the larval genomes [19], [20], [21], [22], [23]. Masaki Kamakura recently found that a specific factor in royal jelly, royalactin, drove queen development through an Egfr-mediated signaling pathway [24].

The worker and queen bee developmental fates can be understood in terms of different development programs that are encoded in the bee genome and have been designed from various components derived from its Hymenopteran ancestry [25]. Analysis of differential genetic expression in anarchic (egg-laying) and wild-type worker bees has led to the suggestion that the queen fate may actually be the default female bee development program [26]. From an evolutionary perspective it also appears reasonable to assume that the queen is closer to the normal insect female, and that the production of a specialized, sterile worker must be a highly costly and very risky strategy that requires tight regulatory control. These observations all seem to imply that it is the worker program that needs to be actively switched on, and, thus, that it is the prospective worker larva that must receive a specific environmental signal (nutritional or other) to activate this program. It appears to be a common assumption that the nurse bee secretions supplied to prospective worker larvae during their first few days of development is royal jelly [2], [27] and thus should be identical in composition to that which the queen larva receives. However, it has been noted that the secretions provided to prospective workers differ in outward appearance and glandular origin from the royal jelly provided to queen larvae [28], and thus the possibility cannot be excluded that nurse bees are capable of differentiating the quality of the glandular excretions supplied to the two types of larvae. Nucleosides corresponding to substantial RNA levels have been isolated from royal jelly, showing that hypopharyngeal glands are capable of secreting these types of molecules [29], [30]. Feeding larvae double-stranded RNA, complementary viral or endogenous mRNAs elicit RNAi responses, thus demonstrating that RNAs in the feed may exert intracellular effects in the larva [31], [32]. MicroRNAs have recently emerged as a class of regulatory molecules endowed with the task of regulating, fine-tuning and maintaining patterns of differential gene expression underlying cellular and tissue fates [33], [34], including aspects involving epigenetic control [35]. Analyses have revealed specific differences in miRNA composition and concentrations between worker and queen bee adults, pupae [36] and larvae, and we therefore investigated the small RNA content in royal and worker jelly. The results show that worker jelly is far more abundant in miRNA types and concentration than royal jelly. Though individual miRNAs commonly fail to elicit distinct phenotypic changes [34], we show that certain miRNAs supplied to the larval feed of prospective queens are capable of altering specific adult morphological characters in the direction of the worker bee. In one case (miR-184) this included a range of characters and was also reflected in substantial changes to the larval mRNA expression pattern.

## Results

### Expression profiling of worker and royal jelly

We collected worker and royal jelly of the Italian honeybee (ZND No.1, *Apis mellifera ligustica*) at 73∼90 hours (4th-day larvae), 97∼114 hours (5th-day larvae), and 121∼138 hours (6th-day larvae) after hatching. After total RNA was extracted and quantified, equal amounts of total RNAs from each of the three sampling days were pooled into worker and royal jelly samples, and the fraction of small RNAs less than 30nt long was retained and sequenced on the Illumina/Solexa high-throughput platform (HTP). After filtering out short (<18 nt) and low quality reads, we were left with 5,919,507 and 6,523,840 small RNA reads from the worker and royal jelly, respectively, which were used for further analysis.

**Figure 1:**
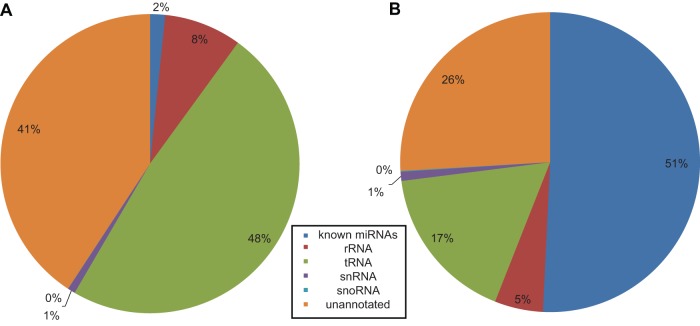
Composition of small RNAs in worker and royal jelly RNA samples.

The most striking difference between the worker and royal jellies is the relative composition of small RNA types. In worker jelly, known miRNAs and tRNAs make up 51% and 17% of the filtered sequence reads, respectively, whereas in the royal jelly, only 2% of the sequence reads represent known miRNAs, compared to 48% representing tRNAs (Figure 1). Other annotated ncRNAs (mainly snRNAs, snoRNAs and rRNAs) made up comparable fractions of the sequences reads (6% and 9% in worker and royal jelly, respectively), whereas sequence reads corresponding to potential transcripts arising from unannotated genomic regions (introns and intergenic regions) constituted 26% and 41% in worker and royal jelly, respectively. Comparison to a parallel analysis in worker and queen bee larvae showed no similar distortion of small RNA category distribution (unpublished data).

### miRNAs are more abundant in worker jelly than in royal jelly

**Figure 2:**
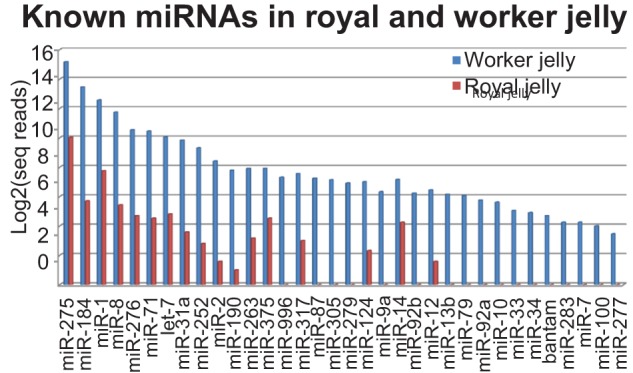
Concentration levels of 34 miRNAs in worker and royal jelly.

There are 58 bee miRNAs annotated by mirBase13.0, and we detected 48 of these in the two jellies (48 in worker jelly and 25 in royal jelly). In terms of sequence reads these 48 miRNAs varied across five orders of magnitude. Fourteen of the known miRNAs had less than 10 sequence reads in both samples, and for these, meaningful concentration differences between the two jellies could not be calculated. For the remaining 34 miRNAs, their concentrations in the worker jelly were invariably much higher (7–215 fold) than in royal jelly (Figure 2, Table S1). Generally, the concentrations of individual miRNAs in royal jelly mirrored those of the worker jelly at lower concentrations (Pearsons correlation coefficient  = 0.967), and the observed differences are more compatible with the notion of overall (or collectively) lower miRNA levels in royal relative to worker jelly, rather than an interpretation in terms of strong expression in individual miRNA levels between two jellies. The distribution of miRNA concentrations in the worker and royal jellies were similar to, but not identical with the miRNA expression patterns observed in worker and queen larvae (Figure S1). In accordance with previous observations in the fly [37] and honey bee [38], we observed passenger strand (miRNA*) sequences for numerous known miRNAs (Table S2), suggesting that such strands are frequently functional in insects [39].

### Novel miRNAs are predominantly found in worker jelly

**Figure 3:**
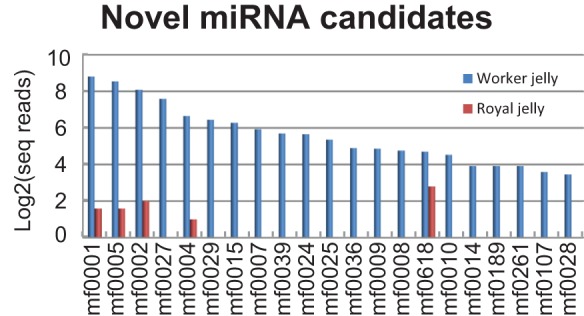
Novel miRNA candidates in worker and royal jelly. Only transcripts represented by more than 10 sequence reads in any sample are shown.

In order to identify possible novel miRNAs, we used the MIREAP software to identify potential stem-loop structures in sequences flanking the remaining unannotated reads. This yielded 44 potential miRNAs derived from 31 miRNA precursors, and further analysis with MiPred [40] confirmed 29 of 31 of these (Table S3). All 44 of the putative miRNAs were detected in worker jelly, and only 6 of these were also seen in the royal jelly sample. Overall, the novel miRNAs were present in far lower concentrations (WJ average ∼47 sequence reads) than known miRNAs (WJ average ∼1240 sequence reads), and only 22 of the novel miRNAs were represented by more than 10 sequence reads in any of the jelly samples (Figure 3). Conservation analysis (mirAlign [41]) showed that among the 31 novel miRNA precursors, five were homologous with miRNA genes in other insects (*Drosophila*) species, and two showed similarity to rodent miRNAs (Table S3).

After removal of novel miRNA candidates and passenger strands, we were left with approximately 30,000 unannotated unique transcripts which could be mapped to 14573 loci. Of these, 7748 and 6050 were found in either royal or worker jelly, respectively, and only 775 loci were represented in both jellies. Based on sequence characteristics and similarities, we could tentatively group some of the unannotated sequence reads into piRNA-, siRNA-, and miRNA-like transcripts (Table S4).

### Jelly miRNAs and their predicted mRNA targets cluster in functionally enriched modules

In order to obtain information on the possible functional roles for the worker and royal jelly miRNAs, we used the Miranda software [42] to predict targets of all known and novel miRNAs detected in the jellies. Manual inspection of the lists of predicted target mRNAs revealed that the 10 miRNAs with the highest concentration in worker jelly (Figure 2) tended to collectively target specific mRNAs. Among the targets of these 10 miRNAs, there were five different mRNAs, each targeted by 6 of the miRNAs. The estimated probability of one mRNA being targeted by 6 miRNAs is approximately 1.8×10^−5^ and the estimated probability of encountering five such cases among the targets is less than 10^−23^. Moreover, each of these five collectively targeted mRNAs is annotated with functions related to central nervous system development (see Table S5). Thus, to identify more modules consisting of groups of co-targeting miRNAs and co-targeted mRNAs, we assembled and used the information on miRNA and mRNA expression profiles to construct a miRNA-mRNA target network. Briefly, we hypothesized that if a miRNA was present in worker jelly at a high concentration (relative to royal jelly), its predicted mRNA targets in worker larvae should be down-regulated relative to queen larvae, and vice versa. On this basis, we selected miRNA-mRNA pairs with correlated variations in jelly miRNA and larval mRNA levels, and used these pairs to construct a “jelly miRNA – bee mRNA” regulatory network with 441 nodes and 2132 edges. From the network, we identified (using the MCODE software [43]) four modules, which we tested for possible functionality by calculating the enrichment of specific Gene Ontology (GO) terms among the mRNAs belonging to each module (Figure S2). All four modules showed significant (p<0.05) enrichment for a number of GO Biological process terms, Cellular component terms and Molecular function terms (Table S6).

### Jelly miRNA contents change dynamically through larval development

**Figure 4:**
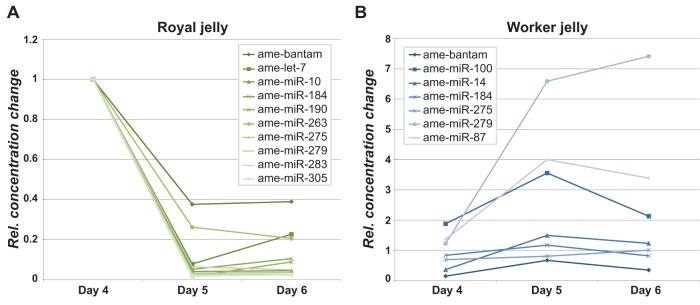
Dynamic variation in jelly miRNA concentrations during larval development. Only miRNAs with significant (p<0.05; t-test) variation in concentration are shown. A. Royal jelly. B. Worker jelly. All concentrations are scaled to 1 on day 4 in royal jelly.

Determination of the characters that distinguish queen and worker bees take place at a particular time points during larval development [25], and it was therefore of interest to monitor the jelly miRNAs during the course of larval development. To this end, we used quantitative RT-PCR to measure the concentrations of 22 known miRNAs in royal and worker jelly collected 4, 5 and 6 days after hatching of the larvae. The qRT-PCR data largely reproduced the differences in royal and worker jelly miRNA concentration. The most consistent trend over the 3-day sampling period was a strong drop in concentration for all tested miRNAs in royal jelly from day 4 to day 5, after which the levels remained stable (Figure 4A). The trend was statistically significant (p<0.05, paired t-test) for 10 of the 22 tested known miRNAs, and among these the decrease in miRNA concentration varied from 2.6 fold (bantam) to 64 fold (miR-279). In comparison, the variations in worker jelly miRNA concentrations were smaller, less systematic, and generally positive, particularly from day 4 to day 5. Worker jelly miRNAs with significant (p<0.05, paired t-test) day-to-day/diurnal changes, either showed a persistent 1.5–1.7 fold increase in concentration from day 4 through day 6 (miR-275 and miR-279), or a transient (1.4–4.5 fold) increase in concentration from day 4 to day 5, followed by a slightly less marked (1.7–2.1 fold) decrease from day 5 to day 6 (*e.g.*, bantam, miR-184; Figure 4B).

In order to validate the qRT-PCR data, and extend the analysis to a wider set of miRNAs and miRNA candidates, we extracted total RNA from fresh royal and worker jelly samples, and hybridized to a microarray composed of 515 probes against almost all isoforms of the 123 detected known and candidate miRNAs detected by the deep sequencing analysis. The microarray data confirmed the general reduction in the miRNA concentrations from day 4 to day 5 and 6 in royal jelly (Figure S3). The worker jelly miRNA concentrations were more variable, with 77 miRNAs showing significant (p<0.01) changes in concentration. Similar to the qRT-PCR data, a majority of these showed either a persistent (28 miRNAs) or transient (15 miRNAs) increase in concentration, while 25 miRNAs had significantly lower concentrations on day 5 than on the preceding and the following day, and only 9 miRNAs showed a persistent fall in concentration through the period (Figure S4). Comparison of royal and worker jelly on individual days gave 8, 17, and 32 miRNAs with significant (p<0.01) differences in concentrations on days 4, 5 and 6, respectively (Table S7). Seven of these miRNAs (miR-12, miR-263, miR-263b, miR-277, miR-283, miR-31a and miR-3), all having lower concentrations in royal jelly, were predicted to target 3 juvenile hormone related genes (GenBank: XM_001119986.1, XM_001121814.1, and XM_396819.3).

### Addition of miRNAs to the larval food influences honeybee morphology

The effects of individual miRNAs on phenotypic characters are usually subtle, and it normally takes a combination of several miRNAs to obtain an observable effect. Single miRNAs should, in most cases, not produce any morphological effect if fed to developing larvae. We nonetheless selected 37 small RNAs that were found in significantly higher concentrations in WJ than in RJ, had them synthesized *in vitro*, and prepared a feeding experiment to test possible effects on bee morphology. To the natural food of 2- and 3-day-old queen larvae was added either 500 ng miRNA dissolved in DEPC-treated water, or DEPC-treated water only (“control”). To enable the observation of possible (negative) effects of the manipulation itself, one of three larvae was left undisturbed, receiving neither water nor miRNA solution (“untreated”). The potential effects of the miRNA supplements were observed on six morphological characteristics (birth weight, body length, proboscis length, wing length, wing width and wing area) of the adult queen bee immediately upon hatching of the pupae. Statistical analysis was carried out with one-way ANOVA and Tukey's test (see Methods).

**Figure 5:**
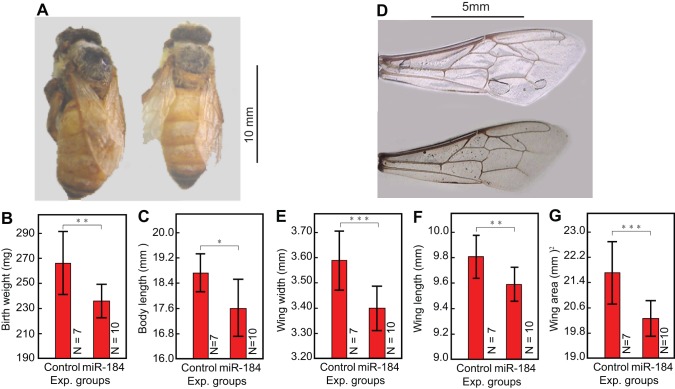
Morphological changes in honeybees treated with miR-184. Comparison between morphological characteristics of newly emerged adults of the miR-184 treated group and the control group: a). the miR-184 treated adult bee (right) and the control adult bee (left); b). the birth weight was significantly reduced by feeding miR-184 in larval food (*F_2,25_* = 5.255, p = 0.0124, One-way ANOVA; p<0.0036, Tukey's test); c). the birth length was significantly reduced by feeding miR-184 in larval food (*F_2,25_* = 4.051, p = 0.0299, One-way ANOVA; P = 0.0285, Tukey's test); d). wings of the miR-184 treated adult bee (Bottom) and the control adult bee (top); e). the wing width was significantly reduced by feeding miR184 in larval food (*F_2,25_* = 8.496, p = 0.0015, One-way ANOVA; P = 0.0003, Tukey's test). f). the wing length was significantly reduced by feeding miR184 in larval food (*F_2,25_* = 4.683, p = 0.0187, One-way ANOVA; P = 0.0055, Tukey's test). g). the wing area was significantly reduced by feeding miR184 in larval food (*F_2,25_* = 9.307, p = 0.001, One-way ANOVA; P = 0.0002, Tukey's test). ***P<0.001; **P<0.01; *P<0.05. See text for details.

As expected, the majority of the tested miRNAs had no significant effect on any of the tested morphological characters. Several small RNAs affected one or more morphological characters of the resulting adult queen bees when supplied in the larval food. The most pronounced effects were obtained with miR-184 which showed a significant influence on all six measured morphological characteristics. The birth weight of newly emerged adults fed with miR-184 was on average 8% lighter than bees that had received DEPC treated water (p<0.0036), and their body length was 5% smaller (p<0.0285; Figure 5). miR-184 also significantly reduced wing width (5%, p<0.0003), wing length (3%, p<0.005) and consequently wing area (7%, p<0.0002) (see Figure 5), but increased the proboscis length of the adult bees (*F_2,31_* = 4.301, p = 0.0225, One-way ANOVA; p = 0.0405, Tukey's test). Other miRNAs only significantly affected one or two of the measured characters. miR-276 reduced wing area (*F_2,38_* = 4.013, p = 0.0262, One-way ANOVA; p = 0.0185, Tukey's test) and proboscis length (*F_2,38_* = 4.344, p = 0.02, One-way ANOVA; p = 0.0211, Tukey's test). Proboscis length was also reduced by miRNA xt0018603 (*F_2,46_* = 4.031, p = 0.0244, One-way ANOVA; p = 0.0198, Tukey's test). Body length was significantly reduced after feeding with miR-33 (*F_2,63_* = 3.664, p = 0.0312, One-way ANOVA, p = 0.0226, Tukey's test), and wing width increased significantly after supplement with miR-12 (*F_2,49_* = 4.102, p = 0.0225, One-way ANOVA; p = 0.033, Tukey's test). Feeding with miR-283 shortened wing length (*F_2,62_* = 2.833, p = 0.0665, One-way ANOVA; p = 0.0496, Tukey's test) and reduced wing area (*F_2,62_* = 5.832, p = 0.0048, One-way ANOVA; p = 0.0065, Tukey's test) significantly.

The results show that a single small RNA appended to the larval food may significantly affect individual morphological characters during the development of the honey bee but will hardly cause major transitions in the overall developmental program. One possible exception to this rule was provided by miR-184 which significantly influenced all measured characters. An overall analysis of all experiments in which this miRNA was tested further gave significant differences in birth weight, body length and wing size between miR-184 treated and control bees, suggesting an overall switch in development towards worker bee differentiation (Table 1). We therefore studied this case further by analyzing the mRNA profiles of queen larvae fed with miR-184.

**Table 1:** Morphological changes in the adult queen after ingestion of miR-184 in royal jelly by the larvae.

	Control Group Mean±SD, n = 57)	Small RNA Group Mean±SD, n = 64)	Normal worker Group Mean±SD, n = 35)
Birth Weight (g)	0.2355±0.02	0.2252±0.0309	0.1249±0.0093
	T-test, P = 0.0333		
Body Length (mm)	18.0737±1.038	17.5825±1.2879	14.1771±0.6361
	T-test, P = 0.0237		
Wing area (mm^2^)	20.4017±1.0491	19.9734±1.0253	18.2169±0.7213
	T-test, P = 0.0251		

*Note: The method for the feeding experiment is described in “Materials and methods”. The Control and miR-184 groups consisted of queen larvae reared with royal jelly in queen cups. These were fed either 5 ul DEPC-treated water (Control group) or 5 ul miR-184 (100 ng/µl) in DEPC-treated water (miR-184 group), respectively, when they were 2 days (26∼32 hrs after hatching) and 3 days (50∼56 hrs after hatching) old. The Normal worker group consisted of worker bees collected from the experimental colony during the same season.*

### Feeding with miR-184 affects the mRNA expression profile of queen larvae

To further study the biological function and potential pathways of microRNAs with RNAi phenotypes, we measured mRNA profiles of bee larvae fed with miR-184 (treated) and water (control). By comparing mRNA profiles of the two groups of bee larvae, we found that there were 279 mRNAs which were differently expressed between treated and control larvae (ratio>2 and p<0.01), of which 200 mRNAs were down-regulated in treated larvae, and 79 mRNAs were down-regulated in controls. GO annotation analysis of these 279 genes showed enrichment for a number of functional and biological process terms (Figure S5). Target prediction with the Miranda package [41] identified 116 potential mRNA targets of miR-184 in the bee genome, of which 64 mRNAs were expressed in the treated or control larvae. Ten of these 64 mRNAs were significantly (p<0.05) upregulated in treated relative to control larvae, and 15 were down-regulated (p<0.05), of which 8 mRNAs showed more than a 2-fold reduced expression in the miR-184 treated larvae. GO analysis 8 mRNAs suggested that these genes were mainly located in the sarcoplasmic reticulum (p<0.05) with molecular functions such as geranylgeranyltransferase activity, GTPase activity, prenyltransferase activity and calcium ion binding (p<0.05).

## Discussion

The results show that the honey bee larval food contains distinct differences in miRNA levels. The data are consistent with a collective (or overall) difference in the levels of all miRNAs between work and royal jelly, rather than with strong specific variation in the levels of individual miRNAs. The miRNA concentrations were highest in worker jelly, and analyses through the course of larval development suggested that the miRNA concentrations in royal jelly fell from a higher level in early larval development to very low levels on days 5 and 6. Several of the miRNAs with the highest and most different concentrations in the worker and royal jelly have the potential to regulate the expression of a number of genes (mRNAs) with essential functions in the honey bee. Moreover, addition of individual miRNAs to the food of queen larvae influences the morphology of the adult bee in the direction of the worker phenotype.

The question of the origin of the jelly RNAs requires further discussion. A number of possibilities exist by which the larval food could be spiked or contaminated with RNAs of bee origin, but none are compatible with the observed differences in overall RNA composition, *i.e.,* a lower level of miRNAs relative to all other small RNAs categories. RNA contamination of jelly from the surroundings (*e.g.*, comb wax, debris from the adult bee population, etc.) is certainly possible, and might – due to the much larger amount of jelly provided to the bee larvae – appear to be diluted in the royal jelly, but the overall RNA composition should be identical in the two samples. A second possibility is that the hypopharyngeal gland secretions contain a spectrum of small RNAs that are accidentally included as a part of the secretion process. Alternatively, RNAs might be included into the secretions in order to supply the young larvae and the queen with a nutritional source of nucleosides [30]. In either case, however, would one expect the RNA composition in both jellies to be similar, and that RNA concentrations in royal jelly would be higher than (or at least equal to) those in worker jelly, since the latter is diluted by additions of pollen and nectar or honey [28]. A third possibility is that the RNAs observed in the larval food are actually contaminations from the larvae themselves. Such contaminations could include secretions from the larvae, cellular debris, or (although very unlikely) even accidental inclusion of entire larvae in the jelly samples. The overall miRNA profiles of the larvae do show some similarity to those of the jellies, but are also different in that worker and queen larvae both specifically express a number of miRNAs that are absent or nearly absent in the other larval type. However, the main argument against a possible contamination from the larvae lies in the overall RNA composition of the two jellies. The sequencing results from worker and queen larvae show nearly identical distributions of the different small RNA categories, and it is not easily explained how contamination from the larvae could possibly produce the differences of small RNA distributions seen in the worker and royal jelly.

Having explored the possibility for a contaminative origin, one is left to consider the possibility that the RNA composition is indicative of a functional role for small RNAs in the larval food. It appears to be a common assumption that the nurse bee secretions supplied to prospective worker larvae during their first few days of development are royal jelly [2], [27], and thus should be identical in composition to that which the queen larva receives. On the other hand, if a similar difference in overall miRNA levels, as that observed between worker and royal jelly, had been seen between two organisms, or between two tissues of the same organism, one would have to assume that miRNA production had been collectively up- or down-regulated in one or the other. Although master switches for miRNA transcriptional control have been suggested [44], collective regulation of the miRNA levels can potentially also be achieved by interference with the post-transcriptional processing and transport systems [45].

Consequently, if the nurse bees are able to identify different receivers of their glandular secretions, selective regulation of the overall miRNA concentrations in the hypopharyngeal secretions is a physiological possibility. Alternatively, as noted in early studies ([28] and refs therein), the secretions provided to worker larvae may differ in glandular origin from those received by the queen bee larvae. In neither case can the possibility be excluded that nurse bees are capable of differentiating the quality of the glandular secretions supplied to the two types of larvae. It has been pointed out that royal jelly bears a certain functional resemblance to other biological structures designed to convey maternal effects to the developing egg cell [30]. The fact that the worker bee has retained a range of the female bee characters [46], including the ability to produce the yolk protein precursor vitellogenin [47], is one such example.

The functional implications of different miRNA levels in the larval food are intriguing. The most highly concentrated jelly miRNAs have the potential, both individually and collectively to influence numerous aspects of early larval development. The miRNA concentrations in worker and royal jelly generally differed by more than 10 fold, which in most cases would effectively extinguish any intracellular effect of the royal jelly miRNAs. The observation that the most abundant miRNAs have the potential to collectively regulate a number of mRNAs, all with functions related to bee central nervous system development, and the fact that all the miRNAs affecting bee morphology when included in the larval food, were among the more abundant worker jelly miRNAs, may both be interpreted in favor of such a view.

In addition to a number of specifically expressed miRNAs, the worker and queen larvae also contain a common set of highly, but differentially, expressed miRNAs. These miRNAs, which are generally expressed at 2–4 fold higher levels in worker relative to queen bee larvae, coincide to a considerable extent in composition and relative expression levels to the miRNAs found in worker jelly (Figure S1). Analysis of mixed-stage honey bee miRNAs suggests that this miRNA complement is among the most highly expressed bee miRNAs [38], and several of these miRNAs are up regulated in nurse bees relative to foragers [48]. If the jelly miRNA set is active in, or required for the maintenance of, the female worker bee genetic program, it would be expressed in the nurse bee, and could thus be transferred from the cytoplasm of the hypopharyngal gland cells to the secreted jellies. Only in the relatively rare situations where queen bee larvae are reared would it be required to specifically reduce the levels of miRNAs in jelly, which could be achieved by temporary reduction of Microprocessor or Exportin5 [49] activity. The observation that the miRNA levels in royal jelly fall through the course of larval development could be explained if the nurse bee hypopharyngeal glands require some time to down-regulate the miRNA secretion after perceiving the presence of queen larvae in the hive.

The question as to how the elevated miRNA concentrations in the worker jelly might contribute to worker larvae caste determination is complicated by the sheer number of detected miRNAs, and this suggests that the jelly miRNAs are likely to exert their function in concert with other miRNAs. We have therefore, in the following, concentrated mainly on a selection of the most abundant worker jelly miRNAs (see Figure 2) which, among other things, appear to collectively regulate a number of mRNAs with functions in the nervous system.

The honey bee is recognized for its advanced mental faculties [50], and given that the worker and queen bee types have very different behavioral characteristics, which to a large extent are innate, and thus must be hard-wired in the brain structures, it is perhaps not so surprising that the most abundant miRNAs in the worker jelly collectively regulate a number of mRNAs related to various aspects of nervous system development. This group of miRNAs, including miR-275, miR-276, miR-1, miR-2, miR-8, miR-184, Let-7 etc., were also shown to be expressed during other insect larval development [51], [52], [53], [54], [55], [56], [57], [58], [59], [60], [61]. A peculiarity of miR-1, which possibly may have relevance to the different feeding regimes for worker and queen bee larvae, is that miR-1 knock-out mutants in *Drosophila* are lethal in 1^st^ larval instar if the larvae are fed (*i.e.,* feeding triggers lethality), but not if starved [56]. miR-184 was the most abundantly expressed miRNA in two mosquito species [62], and the *Drosophila* miR-184 has a critical role in female germline and early embryonic development. Loss of the fly miR-184 induces deficient oogenesis and embryogenesis and complete loss of egg production [63], which accords well with a potential role in differentiation between the fertile queen bee and the infertile worker bee programs. Moreover, miR-184 is also expressed in the central nervous system of both insects and vertebrates [57]. In the mouse, miR-184 participates in a regulatory network that controls the balance between proliferation and differentiation of neural stem cells [64], and its expression is under the control of methyl CpG-binding proteins, thus providing a link to DNA methylation [65]. In *Drosophila*, miR-184 is expressed in embryos, larvae and adults [66], and its expression shows dynamic changes through embryo development, particularly in the central nervous system [66], [67].

In conclusion, we have presented data that are compatible with a role for miRNAs in the larval feed in honey bee caste determination. The most likely origin of the miRNAs are the hypopharyngeal secretions produced by nurse bees, and the data suggest an overall reduction in the entire miRNA complement in royal jelly compared to worker jelly. Interpreted in this fashion, determination of the worker bee caste may, at least in part, owe to a “maternal-like” effect executed by the transfer of miRNAs that are highly expressed in the adult nurses or their hypopharyngeal glands, to the young larvae, which thereby “inherit” both developmental programs and societal roles from their foster mothers. Compared to existing hypotheses on the determination of social caste in the honey bee, this idea furnishes an additional layer of regulatory control to developmental fate decision of the female bee.

## Materials and Methods

### Jelly sample collection, RNA extraction and sequencing

We prepared three healthy colonies of “Zhenongda No.1”- a royal jelly high-yielding breed of *Apis mellifera ligustica* [68] at the Huajiachi campus, Zhejiang University. In each colony, worker jelly (WJ) and royal jelly (RJ) were collected at 4-days (73∼90 hrs after hatching), 5-days (97∼114 hrs after hatching) and 6-days (121∼138 hrs after hatching). The jelly samples were collected in 50 ml tubes and immediately transferred to liquid nitrogen and thereafter stored at −80°C until total RNA extraction.

Total RNAs were extracted from the jellies with Trizol (Invitrogen) as per the manual. For small RNA library construction, total RNA was size fractionated on a 15% Tris-Borate-EDTA (TBE) urea polyacrylamide gel and the 18–30 nt fraction was excised. Small RNAs were eluted in 0.3 M NaCl by rotating the slice at room temperature for 4 hours. The eluted RNAs were precipitated and washed in ethanol and resuspended in ultrapure water. The gel-purified small RNAs were ligated to the 5′ RNA adapter with T4 RNA ligase, and the ligation products were gel fractionated and purified, before ligating to the 3′ RNA adapter. The final ligation products were purified and amplified by RT-PCR. The amplification products were gel fractionated and purified by electrophoresis on a 6% polyacrylamide gel in TBE buffer, and a gel slice corresponding to the amplified library was eluted in 0.3 M NaCl by rotating the mixture at room temperature for 4 hours. The purified PCR products were then precipitated using ethanol, and resuspended in nuclease-free water. The purified PCR products were sequenced on the Solexa sequencing platform.

### Bioinformatics analysis of small RNA data

Low-quality reads and 5′/3′ adaptor sequences were filtered out and discarded before mapping the small RNA reads to the honeybee genome [69] with SOAP [70]. Only perfect matches were accepted and retained for the following analysis.

To identify known miRNAs, we aligned all the small RNA reads to mature miRNAs and miRNA hairpins in miRBase13.0 with blast using an E-value threshold of 0.01. As a miRNA gene could have different isoforms, the sum of all sequence reads corresponding to a miRNA gene was used as a digital measure of the gene expression level.

For other known non-coding RNAs and coding genes, we collected all the non-coding RNAs such as rRNAs, tRNAs, snoRNAs, and snRNAs from NCBI, and Rfam and all coding genes from UCSC, and aligned the small RNA reads to these sequences with blast using an E-value threshold of 0.01. All the remaining unannotated distinct small RNAs were tested for their miRNA-encoding potential with “MIREAP” (https://sourceforge.net/projects/mireap/). Given that a miRNA could have different isoforms, we took the predominant small RNA from “MIREAP” as the novel representative miRNA. The Audic and Claverie test was used to compare small RNA expression differences between worker and royal jelly [71]. The small RNA expression profiling data used for this study are publically accessible through GEO (GSE44853).

### miRNA target prediction

We used miRanda [72] to predict the potential mRNA targets for each miRNA by analysing the mRNA 3′UTR sequences.

### Measuring miRNAs with microarray and quantitative reverse transcription PCR

To measure the miRNA levels in the jellies using qRT-PCR, 2.5 μg of total RNA from each sample (4th-day RJ, 5th-day RJ, 6th-day RJ, 4th-day WJ, 5th-day WJ, 6th-day WJ), were reverse-transcribed in replicates. Quantitative PCR was performed with NCode^TM^ First-Strand cDNA Synthesis and qRT-PCR Kits (Invitrogen) according to manufacturer's protocol (Corbett Research,RGene 6000). The specificity of PCR products was assessed by melt curve. The quantitative results were calculated relative to 4th-day values of royal jelly unless otherwise stated.

Based on the sequencing results, we designed a miRNA-microarray to measure the expression of miRNAs in 4th-day/5th-day/6-day worker jellies and royal jellies respectively. Microarray assay was performed by a commercial service provider (LC Sciences). Hybridization was performed overnight on a μParaflo microfluidic chip using a micro-circulation pump (Atactic Technologies) with 100 μL 6xSSPE buffer (0.90 M NaCl, 60 mM Na_2_HPO4, 6 mM EDTA, pH 6.8) containing 25% formamide at 34°C. Detection was carried out by reading tag-specific Cy3 and Cy5 fluorescence in dual-sample experiments. Hybridization images were collected using a laser scanner (GenePix 4000B, Molecular Device) and digitized using Array-Pro image analysis software (Media Cybernetics).

Data were analyzed by first subtracting the background and then normalizing the signals using a LOWESS filter (Locally-weighted Regression) [73]. For the two colour experiments, the ratio of the two sets of detected signals (log2 transformed, balanced), one way ANOVA for different stage miRNA profiles, and the paired t-test between WJ and RJ were calculated. Data classification involved a hierarchical clustering method using average linkage and Euclidean distance metric and was visualized with TIGR's MeV (Multiple Experimental Viewer). The miRNA-microarray expression profiling data are publically accessible through GEO (GSE50457).

### Feeding of larvae with miRNA solutions

Eight experimental colonies of *Apis mellifera ligustica* ZND No.1 were used. Three colonies were used for reproduction of larvae, and the remaining five were prepared for rearing queens. The larvae were prepared for the experiment 2∼8 hrs after hatching by inserting an empty comb into the brood box of colony at 7∶00, and then transferring the comb with newly-laid eggs to the super box at 13∶00 on the same day. After 72 hrs, the hatched larvae were grafted into plastic cells for queen breeding, which were arranged in a line and adhered onto queen cell frames at 15∶00. Thereafter, the frames were transferred to the queen-breeding colonies. After 24 hrs, we brought the queen cell frames out, and marked the accepted queen cells “1, 2, 3” in sequence (Figure S6), each number denoting an experimental group of 20∼25 queen cells. 26∼32 hrs after hatching, the treatment for the larvae was designated as following: Group 1 was the “untreated group”, Group 2 was fed with 5 µl DEPC-water for each larva as “control group”, and Group 3 was fed with 5 µl 100 ng/µl microRNA as “miRNA-treated group”. DEPC water and microRNA solution were carefully applied between the two ends of the C-shaped larvae by a micro-pipette. After treatment, the queen cell frames were kept in an incubator (34.5°C, 75%RH) for 2 hrs to ensure that larvae had enough time for intake of the microRNAs. 24 hrs later (the larvae were 50∼56 hrs old), the same treatment was repeated. Ten days later, the plastic queen cells were carefully transferred to 10 ml tubes arranged in test tube racks, and kept in the incubator till emergence of the adult. The emerging adults were transferred to 1.5 ml tubes within 6 hrs, then marked and stored at −80°C until further investigation. When all bees treated at the same time had emerged, we examined their morphological features such as birth weight, body length, proboscis length, wing length, wing width and wing area. The data with regard to morphological indexes were arranged and proofread before statistical analysis with One-way ANOVA and Tukey's test of DPS Software (http://www.chinadps.net/index.htm).

### mRNA analysis after feeding bee larvae with miR-184

Total RNAs of RNAi sample and control sample were extracted with Trizol (Invitrogen). Sequence tag preparation was done with Illumina's Digital Gene Expression Tag Profiling Kit according to the manufacturer's instructions. Briefly, the enriched mRNAs were reverse-transcribed into first strand cDNA with biotin-labelled oligo(dT). The second cDNA strand was synthesized with DNA polymerase I and RNase H, and then was completely digested with restriction enzyme Nla III.

The digested products with 3′ poly(A) end were enriched by streptavidin, and then the 5′ end of digested products were ligated with GEX Adaptor 1 which contains the restriction enzyme digestive site of MmeI. The ligated products were digested with MmeI and depleted of the fragments with poly(A). Next, GEX Adaptor 2 was ligated to 3′end of the above enriched fragments. Finally, the ligated products were amplified by PCR, purified (about 85bp) and sequenced by Illumina/solexa platform.

After sequencing, the sequencing reads were aligned against mRNAs and genome sequences of *Apis mellifera* that were downloaded from Genbank and UCSC. The expression level for each gene was measured by summing all the reads which uniquely mapped to the same gene in the corresponding library. The Audic and Claverie test was used to test the statistical significance of expression differences for each mRNA gene between the RNAi sample and Control sample [71]. The mRNA expression profiling data used for miR-184 study are publically accessible through GEO (GSE44911).

## Supporting Information

Figure S1 **Comparison of known miRNA levels in worker bee larvae and worker jelly.**
(PDF)

Figure S2 **Module 1 in the miRNA-mRNA network.**
(PDF)

Figure S3 **Heatmap of miRNA expression of royal jellies in fourth, fifth and sixth day time points.** QRA, QRB and QRC stand for the fourth, fifth and sixth day royal jelly, respectively. QRA-01, QRA-02 and QRA-03 stand for the three replicates.
(TIFF)

Figure S4 **Heatmap of miRNA expression of worker jellies in fourth, fifth and sixth day time points.** WRA, WRB and WRC stand for the fourth, fifth and sixth day worker jelly, respectively. WRA-01, WRA-02 and WRA-03 stand for the three replicates.
(TIFF)

Figure S5 **Enriched Gene Ontology term of 279 differentially expressed mRNAs after feeding larvae miR-184.** A. Molecular function terms. B. Cellular component terms.
(DOC)

Figure S6 **Biological experiments of feeding microRNAs to Honeybee larvae.**
(PDF)

Table S1 **Sequence reads for known miRNAs in royal (QJM) and worker (WJM) jelly.**
(DOC)

Table S2 Passenger strands (miRNA*) of known honey bee miRNAs detected in worker and royal jelly.
(DOC)

Table S3 **Novel honey bee miRNA candidates detected in worker and royal jelly.**
(DOC)

Table S4 **Characterization of unannotated novel transcripts.**
(DOC)

Table S5 **Five genes each predicted to be targets of 6 of the 10 most abundant miRNAs in worker jelly.**
(DOC)

Table S6 **Enriched GO terms in the miRNA-mRNA network modules.**
(DOC)

Table S7 **miRNAs that were differently (p<0.05) expressed in the microarray.**
(DOC)
