# Supplementary figures and images for: Recipe for a Busy Bee: MicroRNAs in Honey Bee Caste Determination
Source: PLoS One. 2013 Dec 11;8(12):e81661. doi: 10.1371/journal.pone.0081661 (PMC3862878; doi:10.1371/journal.pone.0081661)

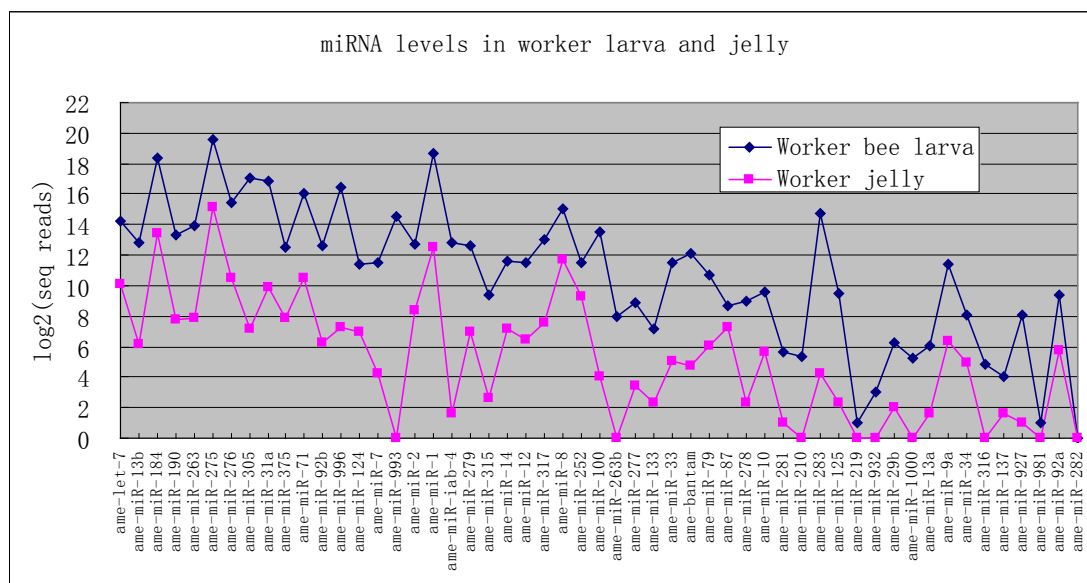

**Supplementary figure S-1. Comparison of known miRNA levels in worker bee larvae and worker jelly.**

Supplement: Figure S1 — Comparison of known miRNA levels in worker bee larvae and worker jelly. (PDF) [file pone.0081661.s001.pdf]

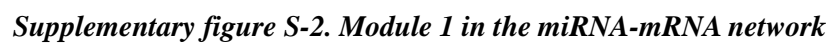

Supplement: Figure S2 — Module 1 in the miRNA-mRNA network. (PDF) [file pone.0081661.s002.pdf]

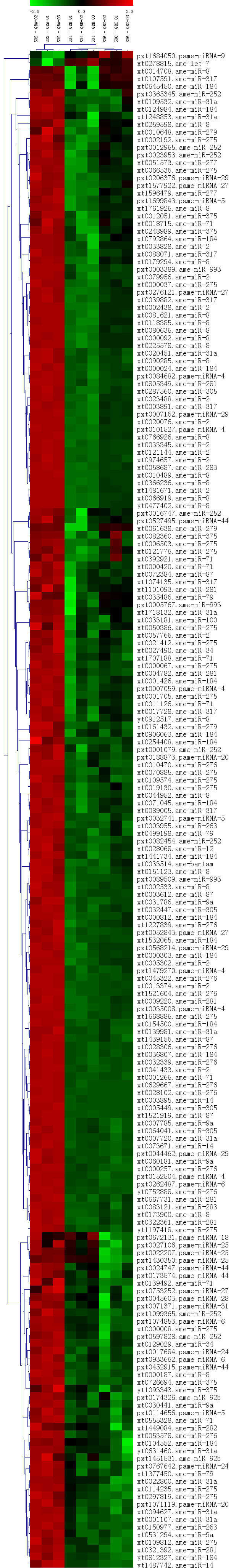

Supplement: Figure S3 — Heatmap of miRNA expression of royal jellies in fourth, fifth and sixth day time points. QRA, QRB and QRC stand for the fourth, fifth and sixth day royal jelly, respectively. QRA-01, QRA-02 and QRA-03 stand for the three replicates. (TIFF) [file pone.0081661.s003.tiff]

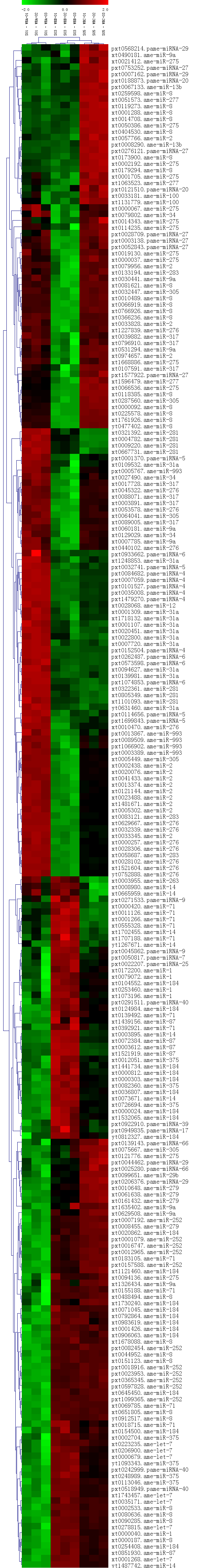

Supplement: Figure S4 — Heatmap of miRNA expression of worker jellies in fourth, fifth and sixth day time points. WRA, WRB and WRC stand for the fourth, fifth and sixth day worker jelly, respectively. WRA-01, WRA-02 and WRA-03 stand for the three replicates. (TIFF) [file pone.0081661.s004.tiff]

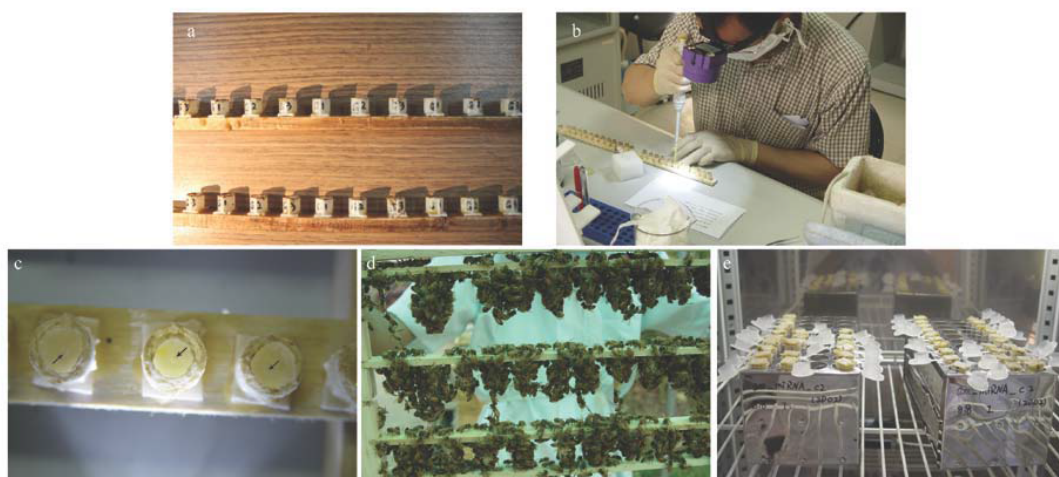

Supplementary Figure S-6: Biological experiments of feeding microRNAs to honeybee larvae

Supplement: Figure S6 — Biological experiments of feeding microRNAs to Honeybee larvae. (PDF) [file pone.0081661.s006.pdf]
