# Supplementary material for: Recipe for a Busy Bee: MicroRNAs in Honey Bee Caste Determination
Source: PLoS One. 2013 Dec 11;8(12):e81661. doi: 10.1371/journal.pone.0081661 (PMC3862878; doi:10.1371/journal.pone.0081661)
Supplement: Figure S5 — Enriched Gene Ontology term of 279 differentially expressed mRNAs after feeding larvae miR-184. A. Molecular function terms. B. Cellular component terms. (DOC) [file pone.0081661.s005.doc]

A.

B.

Supplementary figure S-5

Enriched Gene Ontology term of 279 differentially expressed mRNAs after feeding larvae miR-184. Colored (yellow or orange) circles indicate significantly enriched GO terms, white circles are parent or offspring GO terms. The deeper the color the more statistically significant is the GO term enrichment.

A. Molecular function terms. B. Cellular component terms.
