# Supplementary material for: Recipe for a Busy Bee: MicroRNAs in Honey Bee Caste Determination
Source: PLoS One. 2013 Dec 11;8(12):e81661. doi: 10.1371/journal.pone.0081661 (PMC3862878; doi:10.1371/journal.pone.0081661)
Supplement: Table S1 — Sequence reads for known miRNAs in royal (QJM) and worker (WJM) jelly. (DOC) [file pone.0081661.s007.doc]

Supplementary table S-1. Sequence reads for known miRNAs in royal (QJM) and worker (WJM) jelly. (Audic and Claverie test [1]),

| microRNA ID | WJM count | QJM count | P-value[1] |
| --- | --- | --- | --- |
| ame-miR-184 | 11205 | 52 | 0 |
| ame-miR-275 | 36805 | 1040 | 0 |
| ame-miR-276 | 1485 | 26 | 0 |
| ame-miR-71 | 1396 | 23 | 0 |
| ame-miR-1 | 6047 | 217 | 0 |
| ame-miR-8 | 3432 | 43 | 0 |
| ame-let-7 | 1058 | 28 | 1.41E-294 |
| ame-miR-31a | 917 | 12 | 4.54E-273 |
| ame-miR-252 | 634 | 7 | 2.48E-191 |
| ame-miR-2 | 340 | 3 | 1.85E-104 |
| ame-miR-993 | 286 | 3 | 2.93E-87 |
| ame-miR-190 | 221 | 2 | 3.25E-68 |
| ame-miR-263 | 241 | 9 | 4.84E-65 |
| ame-miR-996 | 158 | 0 | 9.99E-52 |
| ame-miR-375 | 241 | 23 | 5.05E-52 |
| ame-miR-317 | 189 | 8 | 2.99E-50 |
| ame-miR-87 | 152 | 1 | 7.00E-48 |
| ame-miR-305 | 141 | 0 | 3.05E-46 |
| ame-miR-279 | 121 | 1 | 5.63E-38 |
| ame-miR-124 | 128 | 5 | 6.54E-35 |
| ame-miR-14 | 143 | 19 | 1.09E-27 |
| ame-miR-9a | 80 | 0 | 1.47E-26 |
| ame-miR-92b | 75 | 0 | 6.02E-25 |
| ame-miR-12 | 87 | 3 | 1.46E-24 |
| ame-miR-13b | 71 | 0 | 1.18E-23 |
| ame-miR-79 | 67 | 0 | 2.29E-22 |
| ame-miR-92a | 54 | 0 | 3.59E-18 |
| ame-miR-10 | 49 | 1 | 4.01E-15 |
| ame-miR-33 | 33 | 1 | 4.03E-10 |
| ame-bantam | 26 | 0 | 3.89E-09 |
| ame-miR-34 | 30 | 1 | 3.43E-09 |
| ame-miR-283 | 19 | 0 | 7.05E-07 |
| ame-miR-7 | 19 | 0 | 7.05E-07 |
| ame-miR-100 | 16 | 0 | 6.55E-06 |
| ame-miR-277 | 11 | 1 | 0.001959 |
| ame-miR-315 | 6 | 0 | 0.011027 |
| ame-miR-133 | 5 | 0 | 0.0231804 |
| ame-miR-278 | 5 | 0 | 0.0231804 |
| ame-miR-125 | 5 | 0 | 0.0231804 |
| ame-miR-29b | 4 | 0 | 0.0487272 |
| ame-miR-iab-4 | 3 | 0 | 0.102429 |
| ame-miR-137 | 3 | 0 | 0.102429 |
| ame-miR-927 | 2 | 0 | 0.215316 |
| ame-miR-281 | 2 | 0 | 0.215316 |
| ame-miR-13a | 3 | 1 | 0.317236 |
| ame-miR-210 | 1 | 0 | 0.452612 |
| ame-miR-316 | 1 | 0 | 0.452612 |
| ame-miR-282 | 1 | 0 | 0.452612 |
