# Supplementary material for: Recipe for a Busy Bee: MicroRNAs in Honey Bee Caste Determination
Source: PLoS One. 2013 Dec 11;8(12):e81661. doi: 10.1371/journal.pone.0081661 (PMC3862878; doi:10.1371/journal.pone.0081661)
Supplement: Table S2 — Passenger strands (miRNA*) of known honey bee miRNAs detected in worker and royal jelly. (DOC) [file pone.0081661.s008.doc]

*Supplementary table S-2. Passenger strands (miRNA*) of known honey bee miRNAs detected in worker and royal jelly. LEN denotes nucleotide length, WJ and QJ denotes sequence reads in worker and royal jelly, respectively*

| miRNA* name | hairpin name | sequence | LEN | WJ | QJ |
| --- | --- | --- | --- | --- | --- |
| ame-mir-10* | ame-mir-10 | AAATTCGGTTCTAGAGAGGTTT | 22 | 246 | 3 |
| ame-mir-100* | ame-mir-100 | CAAGCTCCTATCTACCGGTACA | 22 | 1 | 1 |
| ame-mir-12* | ame-mir-12 | CAGTACTTGTGTTATACTTACG | 22 | 1 | 0 |
| ame-mir-125* | ame-mir-125 | ACAGGCTAGATTCTCTGGTAT | 21 | 1 | 0 |
| ame-mir-137* | ame-mir-137 | ACGCGTATTCTTGGGGAATTAA | 22 | 1 | 0 |
| ame-mir-13a* | ame-mir-13a | ACATCAAATTGGTTGTGGAAT | 21 | 11 | 0 |
| ame-mir-13b* | ame-mir-13b | TCGTCAAATTGGTTGTGGCGTG | 2.20E+01 | 7 | 0 |
| ame-mir-14* | ame-mir-14 | GGGGGTGAGAAACTGGCTTGGCT | 2.30E+01 | 66 | 5 |
| ame-mir-184* | ame-mir-184 | CCTTATCATTCTCCTGTCCGGT | 2.20E+01 | 8 | 0 |
| ame-mir-2-3* | ame-mir-2-3 | CCATCAAAGTTGGTTTGTCAT | 2.10E+01 | 2 | 0 |
| ame-mir-252* | ame-mir-252 | CTGCTGCTCAAGTGCTTATCA | 2.10E+01 | 1 | 0 |
| ame-mir-275* | ame-mir-275 | CGCGTTACTCGGGTACTTTAGGCT | 2.40E+01 | 23 | 0 |
| ame-mir-276* | ame-mir-276 | AGCGAGGTATAGAGTTCCTACG | 2.20E+01 | 61 | 0 |
| ame-mir-278* | ame-mir-278 | CCGGATGAGGTCTTCATCGAC | 2.10E+01 | 5 | 2 |
| ame-mir-279* | ame-mir-279 | AATGAGTGAAGGTCTAGTGCACA | 2.30E+01 | 28 | 0 |
| ame-mir-281* | ame-mir-281 | AAGAGAGCTATCCATCGACAGTA | 2.30E+01 | 2 | 2 |
| ame-mir-283* | ame-mir-283 | CAGGATTCTTGCTGGTAT | 1.80E+01 | 14 | 0 |
| ame-mir-305* | ame-mir-305 | CGGCACCTGTTGGAGCGCAATTC | 2.30E+01 | 5 | 0 |
| ame-mir-316* | ame-mir-316 | CCAGCAAAGGGGAACAGGCCGA | 2.20E+01 | 15 | 0 |
| ame-mir-317* | ame-mir-317 | AGGGAGCCACTCTGCGTTCACT | 2.20E+01 | 3 | 0 |
| ame-mir-33* | ame-mir-33 | CAATACTTCTACAGTGCAACT | 2.10E+01 | 1 | 0 |
| ame-mir-34* | ame-mir-34 | CGACCGCTATCGGCACTGCAATT | 2.30E+01 | 4 | 0 |
| ame-mir-71* | ame-mir-71 | TCTCACTATCTTGTCTTTCATC | 2.20E+01 | 9 | 0 |
| ame-mir-79* | ame-mir-79 | CTTTGGTAATATAGCTCTATGA | 2.20E+01 | 7 | 0 |
| ame-mir-8* | ame-mir-8 | CATCTTACCGGGCAGCATTAG | 2.10E+01 | 1939 | 24 |
| ame-mir-87-2* | ame-mir-87-2 | GGGCCTGACTCTTTGCTCTGCC | 2.20E+01 | 1 | 0 |
| ame-mir-92b-1* | ame-mir-92b-1 | AGGTCAGGACTAATGCAAATATG | 2.30E+01 | 25 | 0 |
| ame-mir-92b-2* | ame-mir-92b-2 | AGGTCAGGACTAATGCAAATATG | 2.30E+01 | 25 | 0 |
| ame-mir-993* | ame-mir-993 | GAAGCTCGTCTCTACAGGTATC | 2.20E+01 | 117 | 1 |
| ame-mir-996* | ame-mir-996 | GGCGAGTATGAATGTGGTGCACG | 2.30E+01 | 5 | 0 |
| ame-mir-9a* | ame-mir-9a | ATAAAGCTAGGTTACCGGAGTTA | 2.30E+01 | 109 | 4 |
