# Supplementary material for: Recipe for a Busy Bee: MicroRNAs in Honey Bee Caste Determination
Source: PLoS One. 2013 Dec 11;8(12):e81661. doi: 10.1371/journal.pone.0081661 (PMC3862878; doi:10.1371/journal.pone.0081661)
Supplement: Table S3 — Novel honey bee miRNA candidates detected in worker and royal jelly. (DOC) [file pone.0081661.s009.doc]

*Supplementary table S-3. Novel honey bee miRNA candidates detected in worker and royal jelly. LEN denotes nucleotide length, WJ and QJ denotes sequence reads in worker and royal jelly, respectively. MiRAlign indicate suggested conserved pre-miRNA hairpins in other organisms.*

| miRNA name | sequence | LEN | WJ | QJ | MiRAlign | miPred p-value |
| --- | --- | --- | --- | --- | --- | --- |
| pame-miRNA-1 | TTAAGTAGTAGTGTCGTAGATGA | 23 | 446 | 3 | 0 | 0.001 |
| pame-miRNA-5 | TGACTAGAGTCACACTCGTCCA | 22 | 373 | 3 | dme-mir-279 | 0.001 |
| pame-miRNA-2 | AGTTGGAAGTGAGGATCTAGGCA | 23 | 272 | 4 | 0 | 0.007 |
| pame-miRNA-27 | CATCACAGGCAGAGTTCTAGTT | 22 | 193 | 0 | dme-mir-11 | 0.001 |
| pame-miRNA-4 | CCAGATCTAACTCTTCCAGCT | 21 | 101 | 2 | 0 | 0.004 |
| pame-miRNA-29 | TGACTAGATCCACACTCATCCA | 22 | 87 | 0 | dme-mir-279 | 0.001 |
| pame-miRNA-15 | GTAGGCCGGCGGAAACTACTTGC | 23 | 7.80E+01 | 0 | 0 | 0.001 |
| pame-miRNA-7 | TATCACAGCAGTAGTTACCTGGTA | 24 | 6.10E+01 | 0 | 0 | 0.001 |
| pame-miRNA-39 | ATCGGATCAGGATAGGGTA | 19 | 5.20E+01 | 0 | 0 | 0.001 |
| pame-miRNA-24 | TCAGGTACTGAGTGACTCTGAG | 22 | 5.00E+01 | 0 | dps-mir-306 | 0.001 |
| pame-miRNA-25 | AATTGCACTCGTCCCGGCCT | 20 | 4.10E+01 | 0 | rno-mir-92-1 | 0.001 |
| pame-miRNA-36 | AGACGGAGGATGAAACGCGGCG | 22 | 3.00E+01 | 0 | 0 | 0.069 |
| pame-miRNA-9 | TCGGGAAGGTAGTTGCGGCGGATT | 24 | 2.90E+01 | 0 | 0 | 0.001 |
| pame-miRNA-8 | TCGGTAAGCAGAGTATAAGACCTT | 24 | 2.70E+01 | 0 | 0 | 0.001 |
| pame-miRNA-618 | TAATCTCATGCGGTAACT | 18 | 2.60E+01 | 7 | mmu-mir-216 | 0.001 |
| pame-miRNA-10 | AAGTGGAGAAGTGGTCTTTA | 20 | 2.30E+01 | 0 | 0 | 0.021 |
| pame-miRNA-14 | ATACGAAAGACCGCGCGGATGTG | 23 | 1.50E+01 | 0 | 0 | 0.02 |
| pame-miRNA-189 | AAGCTGCCTTTTGAAGGGCAACA | 23 | 1.50E+01 | 0 | 0 | 0.002 |
| pame-miRNA-261 | CCAGCAAAGGGGAACAGGCCGA | 22 | 1.50E+01 | 0 | 0 | 0.019 |
| pame-miRNA-107 | TAGTACGGGCAGTACTGGGA | 20 | 1.20E+01 | 0 | 0 | 0.014 |
| pame-miRNA-28 | TGGTAACTCCACCACCGTTGGC | 22 | 1.10E+01 | 0 | 0 | 0.003 |
| pame-miRNA-31 | CAAAGCAGCTTGGTACCTAACT | 22 | 8.00E+00 | 0 | dps-mir-306 | 0.001 |
| pame-miRNA-20 | TCACAACCTTTTTGAGTGAGCGA | 23 | 8.00E+00 | 0 | dme-mir-307 | 0.002 |
| pame-miRNA-17 | TGGAGCTCTGGCTGTGACTTGTG | 23 | 8.00E+00 | 0 | dme-mir-11 | 0.001 |
| pame-miRNA-40 | ATTGGGTCAGGATAGGGCAGG | 21 | 8.00E+00 | 0 | 0 | 0.001 |
| pame-miRNA-11 | AGGTTGGGATGTGGGCATTATTTG | 24 | 7.00E+00 | 0 | rno-mir-92-1 | 0.001 |
| pame-miRNA-18 | TGACTAGATCGAAATACTCGTCC | 23 | 7.00E+00 | 0 | dps-mir-286 | 0.001 |
| pame-miRNA-21 | TACCCTGTAACGTCCTGAGAC | 21 | 7.00E+00 | 0 | 0 | 0.001 |
| pame-miRNA-3 | GACGGGTGCACTCTGGTATCATG | 23 | 5.00E+00 | 1 | dme-mir-279 | 0.001 |
| pame-miRNA-6 | TGAGATTCACTCCTCCAACTTAC | 23 | 5.00E+00 | 0 | 0 | 0.016 |
| pame-miRNA-23 | TCTGTATGGCTCAGGACGATAC | 22 | 5.00E+00 | 0 | 0 | 0.002 |
| pame-miRNA-12 | TCGACGAGAATTCCGTGACCGGTC | 24 | 4.00E+00 | 0 | 0 | 0.003 |
| pame-miRNA-26 | ATATGAACTCTTATGTACGTG | 21 | 4.00E+00 | 0 | 0 | 0.001 |
| pame-miRNA-38 | TCCCCTGTCCTGTCCCGATAG | 21 | 4.00E+00 | 0 | 0 | 0.001 |
| pame-miRNA-13 | AATCCTGCATCAAGTGCGTT | 20 | 3 | 0 | 0 | 0.001 |
| pame-miRNA-22 | TAAGCGTATAGCTTTTTCCCTT | 22 | 3 | 0 | 0 | 0.001 |
| pame-miRNA-37 | ACAACGAGGGTGAAGATTGCG | 21 | 3 | 0 | 0 | 0.002 |
| pame-miRNA-19 | TGCGAGGCTTTTTTTCAACGATG | 23 | 3 | 0 | 0 | ----- |
| pame-miRNA-16 | ACTCACTCAACCTGGGTGTGATG | 23 | 1 | 0 | dme-mir-307 | 0.002 |
| pame-miRNA-30 | ATCTTGGCACGATTACTTTTCA | 22 | 1 | 0 | 0 | 0.001 |
| pame-miRNA-32 | CTTGTCCTGGTTCATGTAGGGC | 22 | 1 | 0 | 0 | 0.001 |
| pame-miRNA-128 | ATCGTTGGAAAAAAGTCTTGGAGA | 24 | 1 | 0 | 0 | 0.001 |
| pame-miRNA-263 | CGGCTCGTCATCGGGCACTTTG | 22 | 1 | 0 | 0 | 0.001 |
| pame-miRNA-635 | TCCGTCCAACTCCTTTCCGTCT | 22 | 1 | 0 | 0 | 0.001 |
