# Supplementary material for: Recipe for a Busy Bee: MicroRNAs in Honey Bee Caste Determination
Source: PLoS One. 2013 Dec 11;8(12):e81661. doi: 10.1371/journal.pone.0081661 (PMC3862878; doi:10.1371/journal.pone.0081661)
Supplement: Table S4 — Characterization of unannotated novel transcripts. (DOC) [file pone.0081661.s010.doc]

*Supplementary Table S-4. Characterization of unannotated novel transcripts.*

*After removal of miRNA candidates, unannotated small RNA reads which could be mapped to Apis mellifera genome were tentatively classified as piRNA-like, siRNA-like, miRNA-like and ‘plus RNAs’. piRNA-like transcripts were characterized by a uridine (U) residue at position 1 and an adenine (A) residue at position 10. miRNA-like transcripts were characterized by a U at position 1 and U/C/G at position 10. The remaining transcripts were divided into siRNA-like, which are complementary ESTs (antisense), ‘plus-RNAs’, which map to the sense strand of ESTs, ‘unclassifiable’, which did not fill any of the above criteria.*

|  |  |  | Royal jelly | Worker jelly |
| --- | --- | --- | --- | --- |
| Unannotated unique transcripts | | | 31094 | 28230 |
| piRNA-like | |  | 1378 | 2376 |
| siRNA-like | |  | 2277 | 1965 |
| miRNA-like | |  | 3481 | 4711 |
| "plusRNAs" | |  | 4924 | 4270 |
| Unclassifiable |  |  | 19034 | 14908 |
