# Supplementary material for: Recipe for a Busy Bee: MicroRNAs in Honey Bee Caste Determination
Source: PLoS One. 2013 Dec 11;8(12):e81661. doi: 10.1371/journal.pone.0081661 (PMC3862878; doi:10.1371/journal.pone.0081661)
Supplement: Table S5 — Five genes each predicted to be targets of 6 of the 10 most abundant miRNAs in worker jelly. (DOC) [file pone.0081661.s011.doc]

*Supplementary table S-5. Five genes each predicted to be targets of 6 of the 10 most abundant miRNAs in worker jelly.*

*Based on the average number of targets per miRNA (55.8) and the total number potential targets (mRNAs with annotated 3’UTRs; 2507), the average probability of a miRNA targeting an mRNA is p(miR|mR)=55.8/2507=0.222. The probability of 6 of 10 miRNAs by chance targeting a single mRNA is p(6miR|mR)=10!/(10-6)! x [p(miR|mR)]^6^ = 1.84x10^-5^, and the likelihood of finding 5 such cases is [p(6miR|mR)]^5^ = 2.10x10^-24^.*

| Target gene ID | Function | Targeting miRNAs |
| --- | --- | --- |
| NM_001011567.1 | DopR2 Dopamine receptor | ame-let-7  ame-miR-1  ame-miR-184  ame-miR-275  ame-miR-276  ame-miR-31a |
| NM_001011568.1 | Apis mellifera hyperpolarization-activated ion channel (Amih), mRNA | ame-miR-1  ame-miR-184  ame-miR-2  ame-miR-275  ame-miR-31a  ame-miR-8 |
| NM_001011629.1 | Apis mellifera transcription factor mblk-1-like | ame-let-7  ame-miR-1  ame-miR-184  ame-miR-2  ame-miR-275  ame-miR-31a |
| XM_391958 | PREDICTED: Apis mellifera similar to SoxNeuro CG18024-PA, transcript variant 1 (LOC408411), | ame-miR-184  ame-miR-2  ame-miR-275  ame-miR-276  ame-miR-31a  ame-miR-71 |
| XM_623684 | PREDICTED: Apis mellifera similar to ENSANGP00000021813 (LOC408465), synaptobrevin | ame-miR-1  ame-miR-184  ame-miR-2  ame-miR-275  ame-miR-31a  ame-miR-71 |
