# Supplementary material for: Recipe for a Busy Bee: MicroRNAs in Honey Bee Caste Determination
Source: PLoS One. 2013 Dec 11;8(12):e81661. doi: 10.1371/journal.pone.0081661 (PMC3862878; doi:10.1371/journal.pone.0081661)
Supplement: Table S6 — Enriched GO terms in the miRNA-mRNA network modules. (DOC) [file pone.0081661.s012.doc]

*Supplementary table S-6. Enriched GO terms in the miRNA-mRNA network modules.*

| Module 1 | |  |
| --- | --- | --- |
| Cellular component | |  |
| GO-ID | Description | corr p-value |
| 16020 | membrane | 1.73E-02 |
| 5954 | calcium- and calmodulin-dependent protein kinase complex | 1.73E-02 |
| 34706 | sodium channel complex | 1.73E-02 |
| 1518 | voltage-gated sodium channel complex | 1.73E-02 |
| 5770 | late endosome | 4.84E-02 |
|  |  |  |
| Molecular function | |  |
| GO-ID | Description | corr p-value |
| 4099 | chitin deacetylase activity | 4.91E-02 |
| 19003 | GDP binding | 4.91E-02 |
| 5248 | voltage-gated sodium channel activity | 4.91E-02 |
| 16231 | beta-N-acetylglucosaminidase activity | 4.91E-02 |

| Module 2 | |  |
| --- | --- | --- |
| Biological process | |  |
| GO-ID | Description | corr p-value |
| 35284 | brain segmentation | 2.79E-02 |
| 35283 | central nervous system segmentation | 2.79E-02 |
| 7425 | epithelial cell fate determination, open tracheal system | 3.72E-02 |

| Module 3 | |  |
| --- | --- | --- |
| Biological process | |  |
| GO-ID | Description | corr p-value |
| 35220 | wing disc development | 3.61E-02 |
| 46958 | nonassociative learning | 3.61E-02 |
| 77 | DNA damage checkpoint | 3.61E-02 |
| 7223 | Wnt receptor signaling pathway, calcium modulating pathway | 3.61E-02 |
| 7165 | signal transduction | 3.61E-02 |
| 7444 | imaginal disc development | 3.61E-02 |
| 31570 | DNA integrity checkpoint | 3.61E-02 |
| 35567 | non-canonical Wnt receptor signaling pathway | 3.61E-02 |
| 6898 | receptor-mediated endocytosis | 3.61E-02 |
| 8585 | female gonad development | 3.61E-02 |
| 42770 | DNA damage response, signal transduction | 3.61E-02 |
| 3006 | reproductive developmental process | 3.61E-02 |
| 46545 | development of primary female sexual characteristics | 3.61E-02 |
| 7093 | mitotic cell cycle checkpoint | 3.61E-02 |
| 46660 | female sex differentiation | 3.61E-02 |
| 8103 | oocyte microtubule cytoskeleton polarization | 3.61E-02 |
| 23060 | signal transmission | 3.61E-02 |
| 23046 | signaling process | 3.61E-02 |
| 7294 | germarium-derived oocyte fate determination | 3.61E-02 |
| 48675 | axon extension | 3.61E-02 |
| 30706 | germarium-derived oocyte differentiation | 3.61E-02 |
| 30716 | oocyte fate determination | 3.61E-02 |
| 75 | cell cycle checkpoint | 3.61E-02 |
| 23033 | signaling pathway | 3.61E-02 |
| 7265 | Ras protein signal transduction | 3.61E-02 |
| 48608 | reproductive structure development | 3.70E-02 |
| 8406 | gonad development | 3.70E-02 |
| 16325 | oocyte microtubule cytoskeleton organization | 3.70E-02 |
| 30952 | establishment or maintenance of cytoskeleton polarity | 3.70E-02 |
| 30951 | establishment or maintenance of microtubule cytoskeleton polarity | 3.70E-02 |
| 48588 | developmental cell growth | 3.81E-02 |
| 45137 | development of primary sexual characteristics | 3.98E-02 |
| 48468 | cell development | 3.98E-02 |
| 3 | reproduction | 3.98E-02 |
| 22414 | reproductive process | 3.98E-02 |
| 7088 | regulation of mitosis | 4.03E-02 |
| 51783 | regulation of nuclear division | 4.03E-02 |
| 8407 | bristle morphogenesis | 4.18E-02 |
| 48190 | wing disc dorsal/ventral pattern formation | 4.18E-02 |
| 16049 | cell growth | 4.32E-02 |
| 50808 | synapse organization | 4.32E-02 |
| 7450 | dorsal/ventral pattern formation, imaginal disc | 4.33E-02 |
| 7612 | learning | 4.33E-02 |
| 16055 | Wnt receptor signaling pathway | 4.33E-02 |
| 8587 | imaginal disc-derived wing margin morphogenesis | 4.33E-02 |
| 30154 | cell differentiation | 4.33E-02 |
| 7548 | sex differentiation | 4.33E-02 |
| 22416 | bristle development | 4.33E-02 |
| 23052 | signaling | 4.33E-02 |
| 7293 | germarium-derived egg chamber formation | 4.33E-02 |
| 48869 | cellular developmental process | 4.33E-02 |
| 35222 | wing disc pattern formation | 4.33E-02 |
| 48513 | organ development | 4.61E-02 |
| 8361 | regulation of cell size | 4.94E-02 |
|  |  |  |
| Molecular functions | |  |
| GO-ID | Description | corr p-value |
| 19904 | protein domain specific binding | 4.63E-04 |
| 30165 | PDZ domain binding | 4.57E-03 |
| 8426 | protein kinase C inhibitor activity | 4.57E-03 |
| 30291 | protein serine/threonine kinase inhibitor activity | 4.57E-03 |
| 42813 | Wnt receptor activity | 4.57E-03 |
| 19210 | kinase inhibitor activity | 4.57E-03 |
| 4926 | non-G-protein coupled 7TM receptor activity | 4.57E-03 |
| 4860 | protein kinase inhibitor activity | 4.57E-03 |
| 17147 | Wnt-protein binding | 7.31E-03 |
| 5515 | protein binding | 1.15E-02 |
| 19887 | protein kinase regulator activity | 3.21E-02 |
| 19207 | kinase regulator activity | 3.21E-02 |

| Module 4 | |  |
| --- | --- | --- |
| Molecular function | |  |
| GO-ID | Description | corr p-value |
| 900 | translation repressor activity, nucleic acid binding | 3.38E-03 |
| 30371 | translation repressor activity | 3.38E-03 |
| 90079 | translation regulator activity, nucleic acid binding | 3.38E-03 |
| 8187 | poly-pyrimidine tract binding | 3.49E-03 |
| 45182 | translation regulator activity | 3.55E-03 |
| 3727 | single-stranded RNA binding | 3.80E-03 |
| 3730 | mRNA 3'-UTR binding | 3.80E-03 |
| 3729 | mRNA binding | 2.98E-02 |
