# Supplementary material for: Recipe for a Busy Bee: MicroRNAs in Honey Bee Caste Determination
Source: PLoS One. 2013 Dec 11;8(12):e81661. doi: 10.1371/journal.pone.0081661 (PMC3862878; doi:10.1371/journal.pone.0081661)
Supplement: Table S7 — miRNAs that were differently (p<0.05) expressed in the microarray. (DOC) [file pone.0081661.s013.doc]

***Supplementary Table S-7_. miRNAs that were differently (p<0.05) expressed in the microarray***

| *Stage* | *diff-num* | *miRNA* |
| --- | --- | --- |
| *4th* | *8* | *ame-miR-14 ame-miR-184 ame-miR-275 ame-miR-375 ame-miR-71 ame-miR-276 ame-miR-283 pame-miRNA-4* |
| *5th* | *17* | *ame-miR-14 ame-miR-184 ame-miR-275 ame-miR-375 ame-miR-71 ame-miR-252 ame-miR-1 ame-miR-2 ame-miR-263 ame-miR-279 ame-miR-281 ame-miR-305 ame-miR-8 ame-miR-87 pame-miRNA-4 pame-miRNA-27 pame-miRNA-29* |
| *6th* | *32* | *ame-miR-14 ame-miR-184 ame-miR-275 ame-miR-375 ame-miR-71 ame-miR-276 ame-miR-283 ame-miR-252 ame-miR-2 ame-miR-263 ame-miR-279 ame-miR-281 ame-miR-305 ame-miR-8 ame-miR-87 ame-miR-100 ame-miR-12 ame-miR-13b ame-miR-190 ame-miR-277 ame-miR-29b ame-miR-317 ame-miR-34 ame-miR-9a ame-miR-993 ame-bantam ame-let-7 pame-miRNA-20 pame-miRNA-27 pame-miRNA-29 pame-miRNA-4 pame-miRNA-6* |
